# KAP1 silencing relieves OxLDL-induced vascular endothelial dysfunction by down-regulating LOX-1

**DOI:** 10.1042/BSR20200821

**Published:** 2020-08-07

**Authors:** Tianqing Yan, Chang Liang, Haidi Fan, Wei Zhou, Linyan Huang, Suhua Qi, Wan Wang, Ping Ma

**Affiliations:** 1 School of Medical Technology, Xuzhou Key Laboratory of Laboratory Diagnostics, Xuzhou Medical University, Xuzhou City 221004, Jiangsu Province, China; 2 School of Basic Medical Science, Xuzhou Medical University, Xuzhou City 221004, Jiangsu Province, China; 3 Department of Laboratory Medicine, Affiliated Hospital of Xuzhou Medical University, Xuzhou 221002, China

**Keywords:** Endothelial dysfunction (ED), Endothelial nitric oxide synthase (eNOS), KRAB domain-associated protein 1 (KAP1), Lectin-like oxidized low-density lipoprotein receptor-1 (LOX-1), Oxidized low-density lipoprotein (OxLDL)

## Abstract

KRAB domain-associated protein 1 (KAP1) is highly expressed in atherosclerotic plaques. Here, we studied the role of KAP1 in atherosclerosis development using a cell model of endothelial dysfunction induced by oxidative low-density lipoprotein (OxLDL). The phosphorylation and protein levels of KAP1 were similar between OxLDL-treated and non-treated endothelial cells (ECs). KAP1 depletion significantly inhibited the production of OxLDL-enhanced reactive oxygen species and the expression of adhesion molecules in ECs. Treatment with OxLDL promoted the proliferation and migration of ECs, which was also confirmed by the elevated levels of the proliferative markers c-Myc and PCNA, as well as the migratory marker MMP-9. However, these effects were also abrogated by KAP1 depletion. Moreover, the depletion of KAP1 in OxLDL-treated ECs resulted in decreases in the LOX-1 level and increases in eNOS expression. Generally, the data suggest that strategies targeting KAP1 depletion might be particularly useful for the prevention or treatment of atherosclerosis.

## Introduction

Atherosclerosis (AS) is a multifactorial disease, and the cellular and molecular mechanisms underlying AS are unclear. An increasing number of studies have confirmed that atherosclerosis is a chronic inflammatory disease caused by endothelial dysfunction (ED) [1,2]. ED is widely accepted to be an early sign of AS, and oxidized low-density lipoprotein (OxLDL) is thought to be an initial risk factor for ED [3]. Analyses from clinical data have revealed that elevated levels of OxLDL and other oxides in plasma are associated with vascular ED [4,5]. In response to various types of stress, endothelial cells (ECs) may change their functions and initiate the synthesis and release of vasoactive factors, cytokines, or growth factors. These alterations, referred to as ED, contribute to atherosclerotic plaque formation, lesion progression, and ultimately plaque rupture [6].

Lectin-like oxidized low-density lipoprotein receptor-1 (LOX-1), a key scavenger receptor, mediates the binding of OxLDL in vascular cells and induces the activation of signaling pathways involved in the development of oxidative stress and inflammation [7]. LOX-1 mediates OxLDL-induced production of reactive oxygen species (ROS) and adhesion molecules in atherosclerosis [8]. In addition, LOX-1 plays a pivotal role in OxLDL-mediated AS via endothelial nitric oxide synthase (eNOS) uncoupling and nitric oxide (NO) reduction. NO derived from eNOS plays a crucial role in maintaining endothelial homeostasis [9]. ECs incubated with OxLDL (12–24 h) have been shown to induce ED by up-regulating the expression of LOX-1 and inhibiting the function of eNOS [10]. Therefore, the OxLDL/LOX-1 pathway has been implicated in the pathogenesis of ED.

KRAB domain-associated protein 1 (KAP1), also called Triple motif protein 28 (TRIM28) or transcriptional intermediary factor 1 beta (TIF1β), is an essential cofactor of Kruppel-associated box zinc finger proteins (KRAB-ZFPs). KAP1 serves as a transcriptional co-repressor that regulates the transcription of KRAB-ZFP-specific loci by binding to the conserved KRAB domain. Despite the formation of complexes with KRAB-ZFPs, KAP1 also regulates the activity of transcription factors lacking the KRAB domain, such as c-Myc and E2F. KAP1 can also epigenetically modulate chromatin structure by recruiting histone deacetylase, histone methyltransferase, and heterochromatin protein 1 [11]. KAP1 silencing has been shown to down-regulate VEGFR2 expression in ECs exposed to VEGF through the histone methyltransferase KMT5B. Knockdown of endogenous KAP1 inhibits the TNF-α-induced phosphorylation of IKKα/β and IkBα, degradation of IkBα, and nuclear translocation of NF-kB/p65 in ECs [12]. We therefore speculate that KAP1 may also regulate the expression of LOX-1 in ECs after exposure to OxLDL. A recent study has shown that KAP1 is highly expressed in atherosclerotic plaque tissue [13]. These findings suggest that KAP1 may be a novel protein that participates in the pathological mechanisms of AS.

Given that OxLDL-induced ED is considered a pivotal event during the onset of AS and that the OxLDL/LOX-1 pathway has been implicated in the pathogenesis of ED, whether KAP1 plays a role in this process is unknown. Here, we tested the hypothesis that the silencing of KAP1 down-regulates LOX-1 expression during OxLDL-induced ED, thereby mediating the expression of downstream target genes. Our findings suggest that developing targeted strategies to inhibit KAP1 may prove useful in the prevention or treatment of AS.

## Materials and methods

### Cell culture and treatment with OxLDL

EA.hy926 ECs were purchased from the American Type Culture Collection (Rockville, MD, U.S.A.) and were cultured in Dulbecco’s modified Eagle’s medium (DMEM; Gibco, Carlsbad, U.S.A.) with 10% (v/v) fetal bovine serum (FBS; Gibco, Carlsbad, U.S.A.) in a humidified incubator with 5% CO_2_ and 95% air at 37°C. OxLDL was purchased from Yiyuan Biotechnologies (YB-002), Guangzhou, China. Cells were incubated with or without OxLDL (150 μg/ml) for 24 h as previously described [9,14].

### siRNA transfection assay

The siRNA targeting KAP1 (si-KAP1) and negative control siRNA (si-NC) were obtained from GenePharma (Shanghai, China). Human KAP1 siRNA sequences were: 5′-ACAGGACAGAGAACAGAGCTT-3′ (sense) and 5′-GCUCUGUUCUCUGUC-CUGUTT-3′(antisense). The negative control sequences were: 5′-UUCUCCGAAC-GUGUCACGUTT-3′(sense)and 5′-ACGUGACACGUUCGGAGAATT-3′(antisense). The si-KAP1 or si-NC was transiently transfected into EA.hy926 ECs using Lipofectamine 3000 (Invitrogen, Carlsbad, CA, U.S.A.) according to the manufacturer’s protocols. Taking six-well transfection for an example, briefly, cells were transfected when inoculated to 60–80% confluence. Then the medium was discarded, washed with phosphate buffer saline (PBS; NaCl, 137 mmol/l; KCl, 2.7 mmol/l; Na_2_HPO_4_·12H_2_O, 8 mmol/l; NaH_2_PO_4_·2H_2_O, 1.5 mmol/l) for three times and 750 μl Opti-MEM (Gibco, 31985) was added to each well. Next, lipofectamine 3000 reagent and siRNA were diluted with 125 μl Opti-MEM medium, respectively, for 5 min. After that, the two dilutions were mixed for 20 min. Finally, the 250 μl mixture was added into each well. Cells were incubated with transfection complexes for 6 h before the medium was changed. Twenty-four hours later, the cells were used for experiments.

### Measurement of ROS

The ROS detection was performed using the ROS Fluorescent Probe-Dihydroethidium (DHE) Kit (Vigorous, Biotechnology, Beijing). Specifically, 24 h after siRNA transfection, cells were treated with or without OxLDL (150 μg/ml) for another 24 h. Then, the ECs were washed three times with the preheated 37°C Krebs-Henseleit buffer (NaCl, 131 mmol/l; NaHCO_3_, 20 mmol/l; CaCl_2_, 2.5 mmol/l; HEPES, 20 mmol/l; KCl, 5.6 mmol/l; NaH_2_PO_4_, 1 mmol/l; MgCl_2_, 1 mmol/l, glucose, 5 mmol/l, pH = 7.4). Next, a moderate amount of Krebs-Henseleit buffer containing DHE was added into wells. The wells were avoided light and incubated for 30 min. After incubation, cells were washed with preheated Krebs-Henseleit buffer for three times. Finally, images were taken under the Olympus IX73 inverted fluorescence microscope. It was excited with blue or green light and the ROS-positive cells throughout the nuclei were dyed red. The numbers of positive cells from randomly fields were counted under a microscope (Olympus IX73, magnification × 100) and analyzed using Image J 1.25 software (National Institutes of Health, Bethesda, U.S.A.).

### Cell counting Kit-8 (CCK-8) assay

Cell proliferation was detected by CCK-8 assay. Specifically, after transfecting with siRNA for 24 h, EA.hy926 ECs were seeded into 96-well dishes at a density of 1 × 10^4^ cells/well. After adherence, cells were treated with or without OxLDL (150 μg/ml) for another 24 h. Then the CCK-8 reagent (VICMED, VC5001) was mixed with cell culture medium in 1:10, and the mixture was added into each well and incubated for 2 h at 37°C. Finally, the microplate reader (Bio-Tek, Winooski, VT, U.S.A.) was employed to measure the optical density (OD) at 450 nm.

### Cell migration assay

Transwell assay was performed to detect cell migration using transwell chambers (Corning Incorporated, Corning, NY, U.S.A.). Briefly, EA.hy926 ECs transfected with siRNA were digested with trypsin (Beyotime, C0201) and then seeded in the upper chamber (1 × 10^5^ cells/well) with serum-free medium containing OxLDL (150 μg/ml) or not, while 600 μl of medium containing 10% FBS was used as a chemoattractant in the lower chamber. After incubation at 37°C for 24 h, the non-migrated cells on the upper side of the membranes were wiped off with a cotton swab. Migrated cells on the lower side of the membranes were fixed with 4% paraformaldehyde for 15 min and stained with 1% Crystal Violet for 15 min. The numbers of stained cells from randomly fields were counted under a microscope (Olympus IX73, magnification × 200) and analyzed using ImageJ 1.25 software (National Institutes of Health, Bethesda, U.S.A.).

### Western blot analysis

Cells were lysed in RIPA lysis buffer (Beyotime Biotechnology, Shanghai, China) containing 1% protease inhibitor cocktail (VICMED, VPI012), and the cell lysates were then centrifuged at 14000 × ***g*** at 4°C for 15 min. Protein concentrations were quantified using the Bicinchoninic Acid protein assay kit (BCA; Beyotime, P0010). Total proteins (50 μg) were separated by SDS-PAGE and electrotransferred to polyvinylidenedifluoride membranes (PVDF; Millipore, Billerica, MA, U.S.A.). Then the membranes were blocked with TBST buffer (NaCl, 150 mmol/l; Tris, 10 mmol/l; Tween-20 0.05% (v/v) pH 7.6) containing 5% non-fat milk powder at room temperature for 2 h. The membranes were then probed overnight at 4°C with primary antibodies including phospho-KAP1(1:1000, Abcam, ab70369), MMP-9 (1:1000, Abcam, ab38898), KAP1(1:1000, Cell Signaling Technology, #4123), ICAM-1 (1:1000, Cell Signaling Technology, #4915), VCAM-1 (1:500, Affinity, DF6082) and PCNA (1:2000, Servicebio, GB11010), c-Myc (1:1000, Proteintech, 10828-1-AP), followed by incubation with corresponding HRP-conjugated secondary antibodies (1:5000, Proteintech, SA00001-2) at room temperature for 2 h. Proteins on the membranes were visualized by adding the enhanced chemiluminescence reagent (Millipore, U.S.A.). Band intensities were analyzed using ImageJ 1.25 software (National Institutes of Health, Bethesda, U.S.A.) and normalized to that of loading controls.

### Quantitative real-time fluorescent polymerase chain reaction (qRT-PCR)

Total RNA was extracted using the RNAiso Plus(TaKaRa, Code No. 9109) and then reverse-transcribed into cDNA using a Reverse Transcription kit (Takara, Code No. RR036A). Quantitative real-time PCR was carried out by SYBR Green (Takara, Code No. RR820A) using the Roche LightCycler480 system (Roche, Nutley, NJ, U.S.A.). The reactions were performed according to the manufacturer’s protocols and the results were normalized to the Glyceraldehyde-3-phosphatedehydrogenase (*GAPDH*) expression level. The whole melting curves contained single peaks, indicating a specific PCR amplification. The relative amount of each gene was calculated using the 2−ΔΔct as means of duplicate measurements. Supplementary Table S1 lists the primer sequences used in the present study.

### Statistical analysis

All assays were performed at least in triplicate. Data were expressed as the mean ± SEM and analyzed using GraphPad Prism 7 statistical software. Statistical analysis was performed by Student’s *t*-test or one-way analysis of variance (ANOVA) test and *P*<0.05 was considered statistically significant.

## Results

### KAP1 phosphorylation and protein expression were similar between OxLDL-treated and non-treated ECs

**Figure 1:**
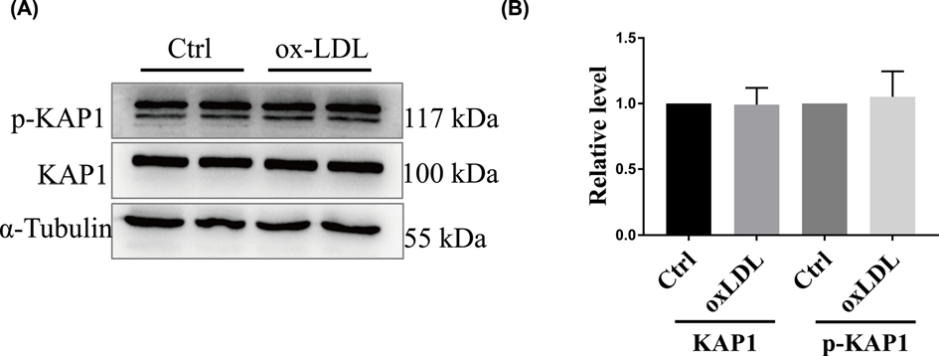
Phosphorylation and protein expression of KAP1 in ECs treated with OxLDL EA.hy926 ECs were treated with or without OxLDL (150 µg/ml) for 24 h. (**A**) Phosphorylation and protein expression of KAP1 were detected using Western blot analysis. Representative blots are depicted from three independent experiments. (**B**) Band intensities were quantified. Data are mean ± SEM.

Here, we first measured KAP1 expression in ED cell models induced by OxLDL. Western blot analysis revealed that the phosphorylation and protein levels were not significantly different between OxLDL-treated and non-treated ECs (Figure 1).

### OxLDL-enhanced adhesion molecular expression and ROS production were diminished by knockdown of KAP1

**Figure 2:**
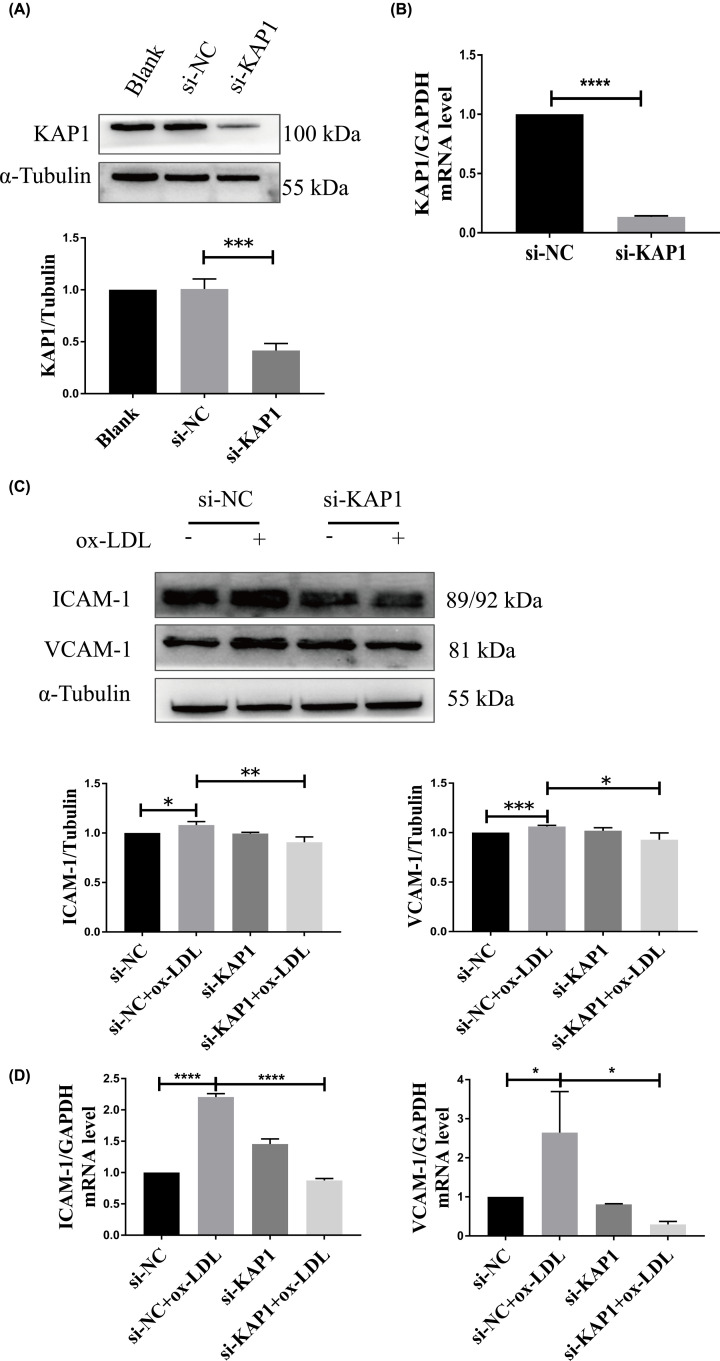
Effects of KAP1 silencing on the expression of adhesion molecules in OxLDL-induced ECs EA.hy926 ECs were transiently transfected with si-KAP1 or si-NC with Lipofectamine 3000 for 24 h. The efficacy of KAP1 knockdown was detected by Western blot analysis (**A**) and qRT-PCR (**B**). Representative blots are depicted from four independent experiments. Band intensities were quantified. Data are mean ± SEM. EA.hy926 ECs transfected with siRNA were treated with or without OxLDL (150 µg/ml) for another 24 h. The expression of ICAM-1 and VCAM-1 was determined using Western blot analysis (**C**) and qRT-PCR (**D**). Representative blots are depicted from three independent experiments. Band intensities were quantified. Data are mean ± SEM (*P<0.05, **P<0.01,***P<0.001, ^****^P<0.0001).

**Figure 3:**
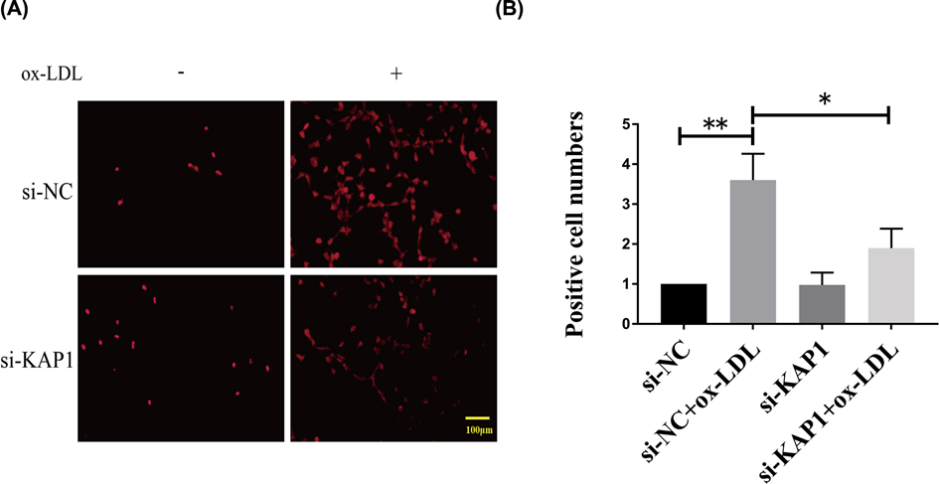
Effects of KAP1 silencing on the generation of ROS in OxLDL-induced ECs EA.hy926 ECs were transfected with either negative control or KAP1 siRNA for 24 h and then treated with or without OxLDL (150 µg/ml) for another 24 h. Next, the DHE fluorescence probe was co-incubated with ECs following the manufacturer’s protocols. (**A**) The ROS-positive cells throughout the nuclei were dyed red under the fluorescence microscope. (**B**) The number of positive cells was quantified. Representative images from three independent experiments are shown. Data are mean ± SEM; scale bars, 100 µm (*P<0.05, **P<0.01).

To further characterize the role of KAP1 in ECs treated with OxLDL, we utilized specific siRNA that could reduce the level of KAP1 in ECs. The efficacy of interference was ascertained by Western blot analysis and qRT-PCR (Figure 2A,B). Next, we measured the expression of pro-inflammatory vascular adhesion molecules, including ICAM-1 and VCAM-1, and found that KAP1 knockdown by siRNA (si-KAP1) significantly abolished the up-regulation of ICAM-1 and VCAM-1 expression in ECs induced by OxLDL (Figure 2C,D). Using DHE staining, we found that OxLDL treatment resulted in sharp intracellular ROS augmentation in ECs. However, KAP1 silencing ameliorated OxLDL-induced cellular ROS production (Figure 3).

### Knockdown of KAP1 inhibited the proliferation and migration of ECs exposed to OxLDL

**Figure 4:**
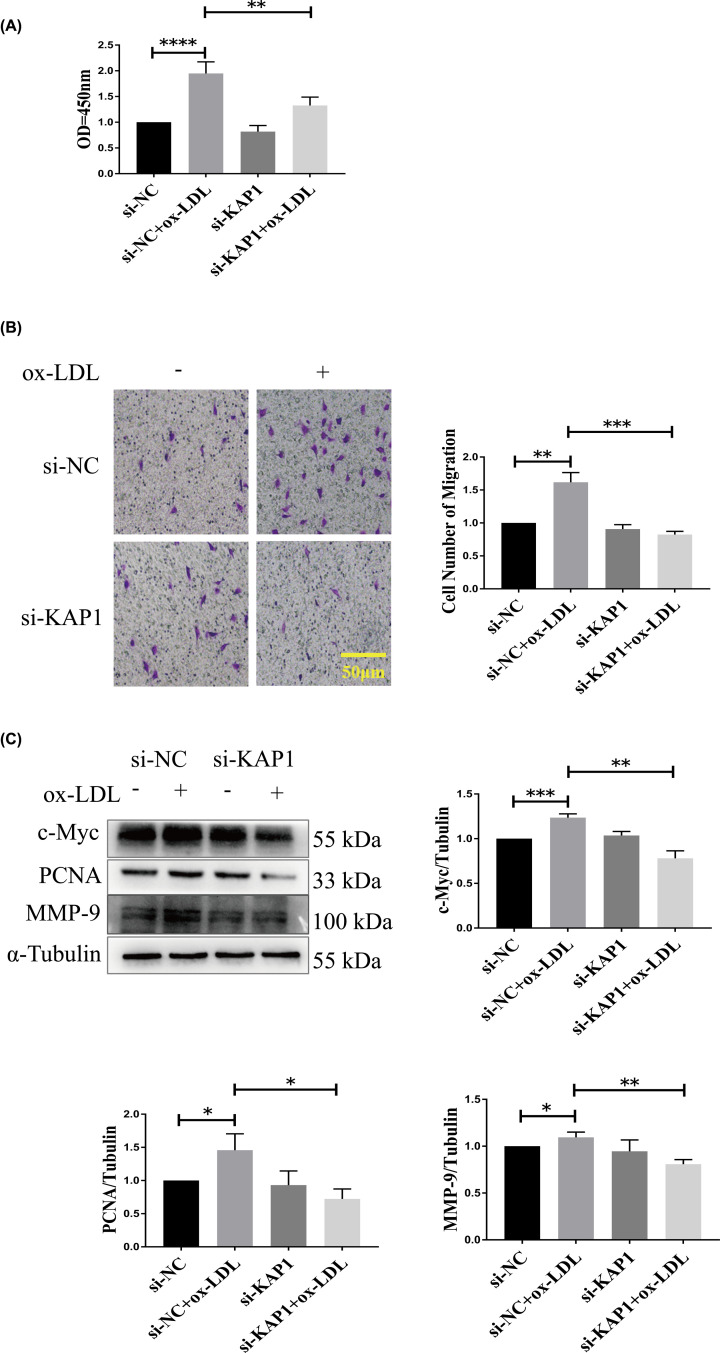
Effects of KAP1 silencing on OxLDL-induced EC proliferation and migration EA.hy926 ECs were transfected with either negative control or KAP1 siRNA for 24 h and then treated with or without OxLDL (150 µg/ml) for another 24 h. (**A**) EA.hy926 EC proliferation was determined by the CCK-8 assay. Data are mean ± SEM. (**B**) Transwell assay was performed to measure cell migration. Representative images from three independent experiments are shown; scale bars, 50 µm. The number of migratory cells was quantified. Data are mean ± SEM. (**C**) Expression of c-Myc, PCNA, and MMP-9 was determined using Western blot analysis. Representative blots are depicted from three independent experiments. Band intensities were quantified. Data are mean ± SEM (*P<0.05, **P<0.01,***P<0.001, ^****^P<0.0001).

We next characterized the role of KAP1 in the proliferation and migration of ECs. CCK-8 and transwell assays revealed that OxLDL-treated ECs demonstrated greater proliferation and migration. However, these alterations were reversed in KAP1 gene-silenced cells induced by OxLDL (Figure 4A,B). In addition, Western blots revealed that si-KAP1 repressed increases in proliferative and migratory markers, including c-Myc, PCNA, and MMP-9 in OxLDL-induced ECs (Figure 4C), further confirming the inhibitory effects of si-KAP1 on the proliferation and migration of ECs stimulated by OxLDL.

### KAP1 silencing significantly inhibited OxLDL-enhanced LOX-1 expression but augmented OxLDL-collapsed eNOS levels in human ECs

**Figure 5:**
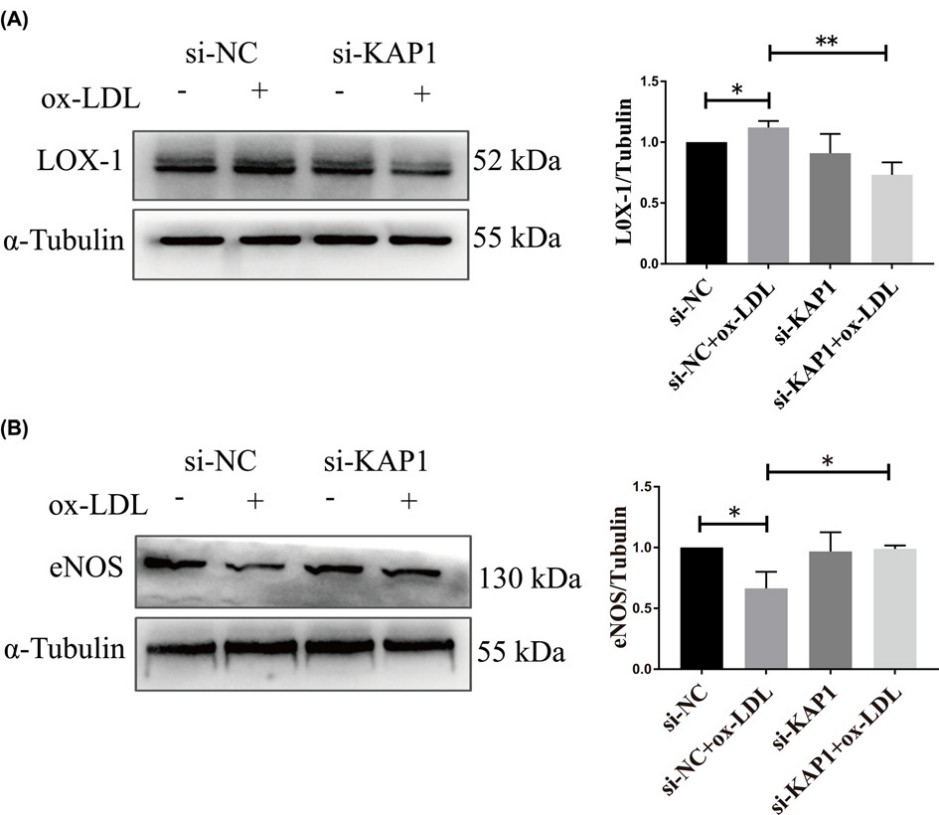
Effects of KAP1 silencing on LOX-1 and eNOS expression in ECs treated with OxLDL EA.hy926 ECs were transfected with either negative control or KAP1 siRNA for 24 h and then stimulated with or without OxLDL (150 µg/ml) for another 24 h. (**A**) Protein levels of LOX-1 were analyzed by Western blotting. Band intensities were quantified. Data are mean ± SEM. (**B**) The expression of eNOS was determined using Western blot analysis. Representative blots are shown from three independent experiments. Band intensities were quantified. Data are mean ± SEM (*P<0.05, **P<0.01).

To determine whether si-KAP1 could affect LOX-1 expression under ED induced by OxLDL, Western blot analysis was performed using specific antibodies targeting LOX-1 and eNOS to demonstrate that the protection provided by KAP1 silencing against OxLDL insult was mediated through the down-regulation of LOX-1. The expression of LOX-1 increased and the expression of eNOS decreased in ECs treated with OxLDL. Nonetheless, KAP1 knockdown significantly reversed these alterations (Figure 5), which may partly explain the potential role of KAP1 in OxLDL-induced endothelium injury.

## Discussion

**Figure 6:**
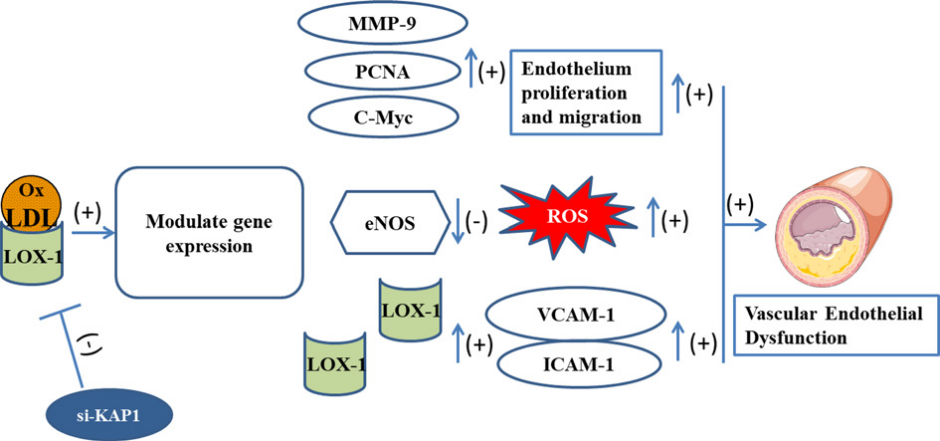
Brief schema illustrating the role of si-KAP1 in ECs Knockdown of KAP1 might inhibit OxLDL-induced ED by suppressing the LOX-1 pathway.

Overall, the results of our study allowed us to construct a brief schema illustrating the role of KAP1 deficiency in ECs. This schema demonstrates that knockdown of KAP1 might counteract OxLDL-induced ED, including the release of ROS, the increase in the expression of adhesion molecules, and abnormal endothelium behavior. Furthermore, this protection is likely correlated with the down-regulation of LOX-1 expression, which leads to the enhancement of antioxidation and the inhibition of aberrant endothelium behavior (Figure 6).

KAP1/TRIM28/TIF1β is a transcription regulatory factor in embryonic development located in the nucleus [12,15,16]. It is widely expressed in multiple tissues and organs, controls stem cell self-renewal, and is involved in chromatin remodeling and the DNA damage response [17]. Numerous studies have shown that KAP1 is highly abundant in malignant tumor cells or tissues, which is associated with the malignant behavior characteristic of the progression of tumors [18–21]. Some studies have also shown that KAP1 is closely related to the regulation of T cells in the immune system [22–24]. However, few studies have examined the role of KAP1 in atherosclerotic cardiovascular and cerebrovascular diseases. Recently, Liu et al. reported that KAP1 was highly expressed in human atherosclerotic tissues and cultured human aortic smooth muscle cells stimulated by platelet-derived growth factor subunit B homodimer [13]. Specifically, they found that knockdown of KAP1 suppressed the proliferation and migration of human aortic smooth muscle cells with atherosclerosis phenotypes, revealing that KAP1 plays a vital role in AS. In the present study, we demonstrated that the phosphorylation and protein level of KAP1 did not differ between OxLDL-treated and non-treated ECs (Figure 1). This pattern may stem from the complicated pathological process of atherosclerosis. After all, the induction of ED by OxLDL accounts for only one small component of the numerous factors contributing to AS. Although some irritants that mimic AS are capable of influencing the expression of KAP1, EC damage caused by OxLDL was not sufficient for inducing any changes in KAP1 phosphorylation and protein expression.

OxLDL-mediated injury to ECs is crucial for ED in the pathogenesis of AS, as well as atherosclerotic plaque rupture at advanced stages [25]. One clinical data analysis showed that serum OxLDL could independently predict pulse-wave velocity in subjects with normal or mildly reduced renal function [4]. Another study revealed that apple polyphenols could inhibit xanthine oxidase, thereby relieving vascular oxidative stress and improving endothelium function [5]. Thus, oxidative stress is closely correlated with cardiovascular and metabolic disease in patients with established renal disease. In addition, OxLDL-induced oxidative stress could activate the NF-κB signaling pathway and promote the expression of adhesion molecules in ECs [26].

In the present study, we showed that ECs treated with OxLDL for 24 h showed increased expression of ICAM-1 and VCAM-1, as well as ROS generation, patterns that are consistent with previous studies [9,26]. However, we found that knockdown of KAP1 suppressed the OxLDL-facilitated expression of ICAM-1 and VCAM-1 (both mRNA and protein levels) and ROS generation in ECs (Figures 2C,D and 3). Thus, KAP1 silencing can protect ECs against OxLDL-induced inflammation and oxidative stress. Moreover, OxLDL-elevated ROS in ECs could stimulate cell proliferation and migration, which is associated with unstable atheromatous plaque [27,28]. In addition, an increasing amount of evidence suggests that inflammation promoted endothelium proliferation and angiogenesis [29]. Cell proliferation and migration were both required for vascular formation, which was correlated with an increased blood supply in atheromatous plaque and resulted in increased sizes of the plaque and blockages in the blood vessels. Thus, abnormal EC behavior plays an important role in contributing to adverse atherosclerotic events. Here, we showed that OxLDL accelerated the proliferation and migration of ECs, while si-KAP1 abrogated this phenomenon (Figure 4A,B). Meanwhile, KAP1 silencing reversed the OxLDL-enhanced expression of two proliferative markers, c-Myc and PCNA, as well as the migratory marker MMP-9 (Figure 4C). Therefore, KAP1 deficiency can improve OxLDL-induced ED via modulation of the abnormal proliferation and migration of ECs, which has a protective function during AS.

Because it is a transcriptional regulatory factor, KAP1 is thought to be associated with the transcription of specific genes and the modification of protein post-translation. KAP1 is known to combine with STATs and interfere with IL-6-mediated signals in HeLa cells [30]. Moreover, KAP1 can inhibit IFN regulatory factor-mediated expression of TNF-α in macrophages [31]. Wang et al. showed that endogenous KAP1 depletion attenuated TNF-α-induced expression of VCAM-1 and ICAM-1 by inhibiting the expression of TNFR-1 and TNFR-2 in ECs [12]. We observed that KAP1 silencing alleviated OxLDL-induced increases in LOX-1 expression (Figure 5A). This finding suggested that the expression of LOX-1 may be regulated by KAP1. The binding of OxLDL to LOX-1 is known to rapidly increase ROS levels in human ECs [8]. Additionally, the activated oxidase stress can further increase LOX-1 levels [32]. The results are consistent with our data. OxLDL/LOX-1-dependent biological processes contribute to plaque instability and the ultimate clinical sequence of plaque rupture along with life-threatening tissue ischemia.

Over the past decades, multiple drugs, including natural antioxidants, anti-LOX-1 antibodies, and antisense oligodeoxynucleotide, have been used to inhibit AS by decreasing vascular LOX-1 expression and activity [25]. Previous studies have noted that the treatment of OxLDL impaired eNOS activity in ECs through LOX-1 activation [33]. In our study, eNOS expression was diminished in ECs after exposure to OxLDL for up to 24 h (Figure 5B). Strategies targeting the enhancement of eNOS activation resulted in increased protection against cell death [34]. Zhou et al. reported that long-term treatment (12–24 h) with OxLDL (100 µg/ml) up-regulated LOX-1 expression and down-regulated the level of eNOS, thereby mediating eNOS activity. Pre-treatment with JTX20 (a LOX-1 blocking antibody) effectively eliminated the ability of OxLDL to mediate the activation of signaling in bovine aortic ECs [35]. Another study has shown that ECs incubated with OxLDL (150 μg/ml) for 24 h markedly increased ROS generation, adhesion molecules and LOX-1 expression but decreased the level of eNOS [14], which is consistent with our findings (Figure 6). Nevertheless, pre-incubation with Klotho (a type of anti-aging protein) effectively prevented OxLDL from affecting any of these alterations. Additional research has revealed that Klotho could alleviate OxLDL-mediated oxidative stress in human umbilical vein ECs via activation of the PI3K/Akt/eNOS pathway and depression of LOX-1 expression [36]. Therefore, LOX-1 represents a promising therapeutic target for the treatment of human atherosclerotic diseases [37].

Although OxLDL-induced ED is considered an important event during AS progression and the OxLDL/LOX-1/eNOS pathway has been implicated in the pathogenesis of ED, there is still a lack of information on whether KAP1 plays a role in this process. In the present study, we tested the hypothesis that si-KAP1 could influence LOX-1 expression during OxLDL-induced ED. KAP1 knockdown significantly down-regulated the expression of LOX-1 but promoted eNOS expression in ECs treated with OxLDL (Figure 5).

Here, we explored the role of KAP1 in OxLDL-induced ED and found that KAP1 silencing protected the endothelium from oxidation damage possibly by inhibiting LOX-1 expression. However, the role of the LOX-1 pathway in mediating the effect of si-KAP1 on ECs remains unclear. Furthermore, other types of cell lines, such as SMCs and macrophages, which are closely associated with atherosclerosis, need to be studied. In addition, an *in vivo* experiment would aid our understanding of the role of KAP1 in the development of AS. Finally, the relationship between KAP1 and the clinical features of AS need to be explored.

In sum, knockdown of KAP1 may protect ECs from OxLDL-mediated injury by depressing the expression of LOX-1 and up-regulating the expression of eNOS, thus participating in the onset and development of AS. Therefore, our study provides new insight into how targeted strategies for depleting KAP1 could be used to control the proatherogenic effects mediated by OxLDL/LOX-1. Specifically, the development of a KAP1 inhibitor or LOX-1 blocker could prove particularly useful for the prevention or treatment of AS.

## Supplementary Material

Supplementary Table S1

## Abbreviations

AS: atherosclerosis
CCK-8: Cell counting kit-8
EC: endothelial cell
ED: endothelial dysfunction
ICAM-1: intercellular adhesion molecule-1
KAP1: KRAB domain-associated protein 1
KRAB-ZFP: Kruppel-associated box zinc finger protein
LOX-1: lectin-like oxidized low-density lipoprotein receptor-1
MMP-9: matrix metalloproteinases-9
OxLDL: oxidized low-density lipoprotein
PCNA: proliferating cell nuclear antigen
ROS: reactive oxygen species
SDS-PAGE: sodium dodecyl sulfate-polyacrylamide gel electrophoresis
TIF1β: transcriptional intermediary factor 1 beta
TRIM28: triple motif protein 28
VCAM-1: vascular cell adhesion molecule-1
