# Supplementary material for: KAP1 silencing relieves OxLDL-induced vascular endothelial dysfunction by down-regulating LOX-1
Source: Biosci Rep. 2020 Aug 7;40(8):BSR20200821. doi: 10.1042/BSR20200821 (PMC7414520; doi:10.1042/BSR20200821)
Supplement: Supplementary Table S1 [file BSR-2020-0821_supp.pdf]

Supplementary Table 1

Primers	Forward(5’-3’)	Reverse(5’-3’)
Human-KAP1	CGCCTTGGGGACAAACAT	CAGTCACCTTCTGGGCATCA
Human-ICAM-1	TCACGGAGCTCCCAGTCCTAA	AAAGGCAGGTTGGCCAATGA
Human-VCAM-1	CGAAAGGCCCAGTTGAAGGA	GAGCACGAGAAGCTCAGGAGAAA
Human-GAPDH	TGTTCGTCATGGGTGTGAACC	GCAGTGATGGCATGGACTGTG
